# Supplementary material for: Hot-Air Drying Significantly Improves the Quality and Functional Activity of Orange Black Tea Compared with Traditional Sunlight Drying
Source: Foods. 2023 May 7;12(9):1913. doi: 10.3390/foods12091913 (PMC10178095; doi:10.3390/foods12091913)
Supplement: Supplementary file 1 [file foods-12-01913-s001.zip › foods-2295441-supplementary.pdf]

Supplementary Materials:

Table S1 Criteria for sensory evaluation of orange black tea

<table>
<tr><th>Item</th><th>Factor</th><th>Quality characteristics</th></tr>
<tr><td rowspan="4">Appearance
10 points</td><td rowspan="4">Color and evenness</td><td>smooth; even color</td></tr>
<tr><td>Still bright and smooth; still uniform</td></tr>
<tr><td>Slightly dark , with spots</td></tr>
<tr><td>Dark color; localized brown spots</td></tr>
<tr><td rowspan="4">Soup color
10 points</td><td rowspan="4">Color</td><td>Red, orange-red, bright</td></tr>
<tr><td>Orange-red, still bright</td></tr>
<tr><td>orange-red dim</td></tr>
<tr><td>Light orange red, dim</td></tr>
<tr><td rowspan="8">Aroma
30 points</td><td rowspan="4">Aroma</td><td>Fruity and pleasant aroma</td></tr>
<tr><td>Fruity and still pleasant</td></tr>
<tr><td>Weak fruity aroma</td></tr>
<tr><td>Unpleasant aroma</td></tr>
<tr><td rowspan="4">Coordination</td><td>Good coordination, both tea and fruity aromas are evident</td></tr>
<tr><td>Coordination, tea or fruity aroma</td></tr>
<tr><td>Less coordinated.</td></tr>
<tr><td>Incongruous, strange aroma</td></tr>
<tr><td rowspan="20">Taste
50 points</td><td rowspan="4">Sour</td><td>Not sour</td></tr>
<tr><td>Slightly sour</td></tr>
<tr><td>Relatively sour</td></tr>
<tr><td>Sour</td></tr>
<tr><td rowspan="4">Sweet</td><td>Sweet and mellow</td></tr>
<tr><td>Relatively sweet</td></tr>
<tr><td>Slightly sweet</td></tr>
<tr><td>Not sweet</td></tr>
<tr><td rowspan="4">Bitter</td><td>Not bitter</td></tr>
<tr><td>Slightly bitter</td></tr>
<tr><td>Bit bitter</td></tr>
<tr><td>Bitter</td></tr>
<tr><td rowspan="4">Astringency</td><td>Not astringent</td></tr>
<tr><td>Slightly astringent</td></tr>
<tr><td>Bit astringent</td></tr>
<tr><td>Astringent</td></tr>
<tr><td rowspan="4">Coordination</td><td>Good coordination of tea and orange flavors</td></tr>
<tr><td>Coordination, the tea taste is obvious,</td></tr>
<tr><td>Less coordinated, the orange taste is obvious</td></tr>
<tr><td>Incongruous, with obvious orange flavor and strong irritation.</td></tr>
</table>

Table S2 Sensory evaluation results of orange black tea with different drying methods

Treatment	Appearance	Soup color	Aroma		Taste				
			Comment	Aroma coordination	Acid	Sweet	Bitter	Astringent	Taste coordination
Hot air drying	Yellow green , still glossy	Red, still bright	Obvious orange fragrance	Moderate coordination	Slightly sour	Sweeter	Slightly bitter	Not astringent	Moderate mellow
Sun drying	Orange yellow, still glossy	Red and bright	Strong medicinal fragrance	More coordinated	Slightly sour	Sweeter	Slightly bitter	Slightly astringent	Slightly bitter

Table S3 Effects of drying methods on volatile components of orange black tea (μg/g)

RT	RI	Compound Name	Hot air drying	Sun drying
5.59	937	α-Pinene	26.1±0.32	25.7±2.52
6.03	963	Benzaldehyde	2.08±0.25	0.98±0.13
6.46	975	Sabinene	36.28±4.77	31.55±4.68
6.83	989	β-myrcene	73.82±4.98	63.19±8.85
7.2	1004	octanal	61.88±4.6	117.39±12.15
8.03	1037	limonene	1168.18±122.8	1120.91±15.58
8.41	1045	Phenylacetaldehyde	13.23±1.57	3.51±0.18
8.48	1295	perillyl alcohol	5.21±0.6	8.76±0.84
8.5	1047	α-Ocimene	0.82±0.11	3.41±0.15
8.9	1059	γ-Terpinene	6.72±0.34	7.57±0.36
9.28	1071	1-Octanol	24.55±1.94	23.51±1.82
9.86	1087	(E)-linalool oxide (furanoid)	23.99±2.99	32.57±4.1
10.37	1104	Linalool	218.58±13.21	123.22±10.46
10.61	1106	Nonanal	23.68±1.36	59.6±5.11
10.87	1113	Phenylethyl alcohol	18.73±1.24	52.41±6.03
11.28	1123	(E)-para-2,8-1-menthadienol	37.32±4.33	24.2±2.23
11.96	1138	(E)-verbenol	43.62±5.07	50.87±6.45
12.26	1145	isopulegol	3.08±0.07	7.11±0.31
12.52	1150	Camphor	-	2.77±0.12
12.72	1154	Citronellal	8.48±0.47	17.81±0.75
13.6	1171	(E)-linalool oxide (pyranoid)	3.25±0.05	4.35±0.08
13.86	1176	linalool oxide (pyranoid)	37.92±2.31	35.29±4.13
14.45	1188	isocarveol	12.3±1.11	12.12±1.62
14.57	1190	Methyl salicylate	54.53±6.85	55.09±1.22
14.81	1195	(-)-α-Terpineol	77.34±7.53	69.16±6.28
15.05	1198	(-)-trans-Isopiperitenol	35.11±2.52	36.88±2.39
15.38	1206	Decanal	96.91±7.38	91.66±8.9
15.63	1210	Octyl acetate	4.24±0.5	4.33±0.29
15.92	1217	(E)-carveol	23.2±1.73	25.72±2.05
16.04	1224	Nerol	31.39±4.39	30.58±4.01
16.35	1228	cis-Carveol	61.7±6.51	65.99±0.64

16.65	1229	Bicyclo[3.1.1]hept-3-en-2-one,4,6,6-trimethyl-, (1R,5R)-	10.29±1.2	8.48±0.34
17.03	1237	(Z)-citral	67.63±8.16	67.05±4.45
17.4	1241	D-carvone	37.33±4.48	40.59±1.45
17.89	1253	Geraniol	52.94±6.97	30.15±4.11
18.57	1264	2-Methyldodecane	2.23±0.12	2.84±0.22
18.8	1268	citral	108.45±11.43	53.7±2.42
19.19	1274	dl-Perillaldehyde	41.12±3.68	68.85±4.85
20.04	1288	2-(4-methylidenecyclohexyl)prop-2-en-1-ol	19.8±1.19	31.17±2.3
21.11	1306	Undecanal	17.52±1.07	24.81±3.28
21.25	1309	2,6,10,10-Tetramethyl-1-oxaspiro[4.5]dec-6-ene	0.97±0.14	3.41±0.08
21.56	1315	2,4-Decadienal	1.98±0.24	5.99±0.36
21.78	1320	(E)-methyl geranate	14.69±0.57	10.33±0.55
22.96	1344	α-Cubebene	21.31±2.71	36.94±2.12
23.29	1350	citronellol acetate	11.61±0.49	22.88±1.16
23.73	1358	Neryl acetate	11.7±1.52	11.51±1.1
23.97	1362	2-(2-Butoxyethoxy)ethyl acetate	2.48±0.37	6.55±0.17
24.16	1366	Decanoic acid	2.1±0.05	3.23±0.42
24.36	1370	α-Copaene	30.32±3.44	30.07±1.79
24.75	1377	acetic acid geranyl ester	23.17±2.68	19.5±1.49
24.84	1379	(3Z)-3-Hexen-1-yl hexanoate	4.29±0.36	2.89±0.28
25	1381	β-cubebene	11.78±1.16	13.87±1.11
25.09	1383	β-elemene	13.35±0.99	13.86±0.5
25.22	1385	cis-Jasmone	4.04±0.55	4.11±0.47
26.13	1401	perillyl acetate	3.41±0.48	4.24±0.29
26.24	1404	Lauryl aldehyde	42.38±5.82	47.07±6.6
26.41	1407	isocaryophyllene	13.26±1.45	7.97±1.03
26.62	1412	α-ionone	0.78±0.12	0.97±0.12
26.92	1418	β-copaene	12.5±0.85	12.57±0.95
27.28	1418	cedrene	3.18±0.38	1.91±0.22
27.59	1431	dihydro-beta-ionol	0.86±0.04	1.17±0.17
27.83	1438	neryl acetone	1.77±0.14	1.88±0.14
28.1	1441	α-Caryophyllene	7.43±0.53	5.48±0.55
28.27	1444	cis-β-Farnesene	16.93±2.17	11.94±0.83
29.04	1459	α-elemene	2.89±0.15	2.58±0.34
29.21	1462	alloaromadendrene	6.55±0.96	3.72±0.21
29.45	1467	β-ionone	20.51±2.56	11.35±1.25
29.89	1475	α-selinene	3.82±0.48	2.25±0.26
30.01	1477	γ-muurolene	2.75±0.33	1.84±0.26
30.23	1481	(-)-β-chamigrene	11.54±1.33	6.38±0.5

30.48	1486	α-amorphene	6.97±0.98	6.16±0.28
31.03	1495	farnesene	23.42±3.29	16.1±1.26
31.29	1500	cubebol	1.07±0.15	1.34±0.02
31.5	1505	(+)-Δ-cadinene	43.33±5.99	32.54±2.52
31.63	1507	calamenene	2.63±0.29	4.16±0.08
31.85	1513	β-Bisabolene	2.48±0.34	2.06±0.14
32.15	1519	Cubenene	4.91±0.56	5.06±0.19
32.53	1527	α-calacorene	1.99±0.26	5.16±0.42
32.96	1537	α-elemol	17.39±2.18	5.25±0.5
33.74	1554	Nerolidol	7.14±1.05	6.48±0.58
34.18	1562	Spathulenol	1±0.15	7.02±0.8
34.36	1566	Nerolidol	2.53±0.36	3.91±0.23
34.75	1574	Globulol	1.82±0.23	1.87±0.12
35.42	1590	cedrol	1.21±0.1	1.78±0.17
35.71	1593	Hexadecane	0.25±0.03	-
36.45	1615	1-epi-cubenol	6.2±0.8	10.03±1.35
36.61	1620	γ-eudesmol	2.98±0.21	-
36.99	1636	T-muurolol	1.93±0.26	2.49±0.3
37.06	1638	T-cadinol	2.21±0.29	5.21±0.25
37.36	1646	(+/-)-agarospirol	8.99±1.01	7.98±0.98
37.7	1658	patchouli alcohol	2.11±0.3	2.36±0.33
37.98	1667	(-)-methyl jasmonate	0.55±0.05	-
38.18	1674	Heptanal, 2-benzylidene-		3.71±0.53
38.5	1685	β-sinensal	23.79±2.75	15.26±1.67
39.69	1741	α-sinensal	24.67±2.77	13.38±1.56
39.92	1754	(Z)-hexadec-9-enal	8.02±0.9	1.88±0.1
40.91	1811	1-hexadecanal	0.6±0.06	0.92±0.11
41.11	1825	farnesoic acid	2.41±0.21	1.74±0.13
41.27	1837	Hexahydrofarnesyl acetone	0.64±0.06	5.5±0.66
41.51	1855	CIS-11-HEXADECENAL	1.98±0.25	0.96±0.04
42.42	1921	Methyl palmitate	0.22±0.03	1.64±0.23
42.83	1956	Palmitic acid	0.62±0.09	0.81±0.08
44.54	2105	Phytol	1.47±0.17	2.13±0.31
		total	3103.465	3019.193

Table S4 Sensory evaluation results of Orange Black tea with different drying temperatures

Treatment	Appearance	Soup color	Aroma		Taste				
			Comment	Aroma coordination	Acid	Sweet	Bitter	Astringent	Taste coordination
40℃	Green yellow brown, still glossy	Red, still bright	Obvious orange fragrance	Moderate coordination	Slightly sour	Sweeter	Not bitter	Not astringent	Moderate mellow

45℃	Green brown, still glossy	Red and bright	Obvious orange fragrance	More coordinated	Slightly sour	Sweeter	Slightly bitter	Not astringent	Moderate mellow
50℃	Green brown, still glossy	Red, still bright	Obvious orange fragrance	More coordinated	Slightly sour	Sweeter	Slightly bitter	Slightly astringent	Moderate mellow
60℃	Yellow brown, still glossy	Red, still bright	Obvious tea fragrance	More coordinated	Slightly sour	Sweeter	Slightly bitter	Slightly astringent	Slightly bitter
